# Automated Building Monitoring System Based on Reflectorless Measurements: A Case Study of the IMSGeo System

**DOI:** 10.3390/s25175327

**Published:** 2025-08-27

**Authors:** Maria E. Kowalska, Janina Zaczek-Peplinska, Sławomir Łapiński, Łukasz Piasta

**Affiliations:** 1 Faculty of Geodesy and Cartography, Warsaw University of Technology, pl. Politechniki 1, 00-661 Warsaw, Poland; janina.peplinska@pw.edu.pl (J.Z.-P.); slawomir.lapinski@pw.edu.pl (S.Ł.); 2 GEOalpin sp. z o.o., ul. Kolektorska 12 lok. 1, 01-692 Warsaw, Poland

**Keywords:** IMSGeo, automatic monitoring, terrestrial laser scanning, reflectorless measurements, data processing

## Abstract

**Highlights:**

**What are the main findings?**

- The IMSGeo system demonstrated high measurement repeatability and accuracy across five surface types, with maximum point coordinate differences not exceeding 5 mm and a surface change detection accuracy reaching up to 97%.
- The use of normal vectors for surface change analysis proved more reliable than direct point-to-point comparisons, especially for unsignaled, reflectorless measurements.

**What is the implication of the main finding?**

- The IMSGeo system enables cost-effective, automated, and reliable geodetic monitoring in real-time, even under varying surface conditions and without the need for signalized targets.
- Proper configuration of measurement parameters, such as point density and field size for normal vector analysis, is crucial for accurately detecting structural deformations and minimizing false alerts.

**Abstract:**

Automatic geodetic monitoring systems allow for real-time monitoring of an object’s condition. The article presents the IMSGeo system (Intelligent Monitoring System for Threatened Objects based on Automatic Non-invasive Measurements), which meets three fundamental efficiency criteria of a monitoring system: reliability, affordability, and the clarity of interpreted results. In this system, the surface is measured using reflectorless methods, and surface changes are determined based on the analysis of normal vectors. The studies were carried out for five typical surfaces: concrete, expanded polystyrene, tiles, brick, and metal. The experiment included two key aspects: analysis of measurement repeatability within accepted accuracy limits and analysis of geometry change determination using a proprietary algorithm. In the first case, a direct comparison of points was made using threshold alerts depending on the repeatability of the measurement. The differences generally did not exceed 5 mm. In the second case, the results showed that the maximum differences for brick and metal surfaces did not exceed 2 mm. For the polystyrene-covered surface, differences for 89% of measurements did not exceed 2 mm; for the tiled surface, 84% did not exceed 2 mm; and for the concrete surface, 97% did not exceed 5 mm.

## 1. Introduction

The monitoring of engineering structures is a crucial aspect of the construction and operation of buildings. With the advancement of measurement technologies, monitoring provides increasingly diverse data and aims for full automation. Interest in ensuring the safety of engineering structures dates back to ancient times when the first large engineering structures were built [1]. An important step in geodesy was the introduction of robotic total stations (RTS) with automatic aiming technologies in the early 1990s. These stations not only increased the manual performance of surveyors in the field but also enabled fully automatic and permanent monitoring installations [2]. Currently, automatic monitoring is used to ensure the safety of key infrastructure objects such as mines [3], bridges [4], threatened buildings, dams [5,6], and underground tunnels [7,8,9]. Monitoring the behavior of engineering structures is an interdisciplinary task. Assessing the safety of structures and preventing the risk of failures and construction disasters requires combining various measurement techniques, computational methods, and the expertise of specialists from different fields of engineering (geotechnicians, surveyors, geologists, structural mechanics, concrete specialists, building materials experts, and many others). Integrating measurements, using numerical modeling of object behavior, and diverse qualitative data allow for a more comprehensive assessment of structures [10,11].

Monitoring is the process of systematic data collection obtained through observation and measurement for further processing and analysis of the results. Careful examination and proper interpretation of the results enable an assessment of the variability of the recorded parameters [3]. The development of measurement technologies creates greater opportunities for more accurate monitoring of engineering structures’ changes and minimizes gross errors. In monitoring systems for threatened objects, it is also important to reduce the time required for data acquisition and personnel involvement, as well as to enable “on-demand” measurements and data transmission in near real-time [12].

Creating a fully automated system that operates in various weather conditions requires addressing numerous technical challenges related to measurement planning and execution, as well as ensuring reliable wireless communication for system control and data collection. The key elements of automatic systems are data processing algorithms and result visualization. The effectiveness of such a system depends on its reliability, affordability, and the clarity of interpreted results. The IMSGeo system (Intelligent Monitoring System for Threatened Objects based on Automatic Non-invasive Measurements), developed by the consortium of GEOalpin Ltd. and Warsaw University of Technology, meets these four fundamental criteria.

Monitoring can be conducted for various purposes: protection of neighboring buildings, assessment of the dynamics of changes taking place, verification of technical condition, prevention and identification of potential threats. It can be either fully automated, partially automated, or entirely manual and use different measurement techniques: tachymetry (angle-linear measurements) [10], global navigation satellite systems (GNSS) [13], terrestrial laser scanning (TLS) [14], unmanned aerial vehicles (UAV) [15], videography [16], inclinometers [17,18], and various sensors in structural health monitoring systems (SHM) [19]. In the study by Pasternak et al. [20], the authors compared four different measurement techniques for assessing the condition of a municipal landfill: unmanned aerial vehicles (UAV), airborne laser scanning (ALS), terrestrial laser scanning, and angle-linear measurements. This comparison indicates that the best solution for monitoring engineering structures with high safety risks in the event of failure is the automation of classical geodetic measurements and the integration of geodetic displacement measurement systems with SHM (physical, geotechnical, hydrotechnical, seismic sensors, and sensors recording vibrations related to the structure’s operation and its surroundings).

Based on the literature, it can be concluded that monitoring systems for objects at risk will differ not only in the measurement techniques and sensors used but also in the value of the algorithms for processing and analyzing measurement data. Examples of leading systems for monitoring endangered objects include: GOCA [21], Automated System of Geodetic Monitoring (Lviv Polytechnic National University, Ukraine) [22], Leica GeoMos [23], Delta LINK (Topcon) [24], Trimble 4D Control [25]. It should be emphasized that GOCA, Automated System of Geodetic Monitoring and IMSGeo are carried out as research and development projects. The remaining systems are commercial solutions. The method of determining changes in an object based on reflectorless measurements obtained from automated motorized total stations is a key element that distinguishes the IMSGeo system from existing solutions on the market [12]. The use of automatic reflectorless measurements significantly reduces monitoring costs, as there is no need to install and maintain reflector prisms on the structure. For users of the monitoring system, it is important that it operates in real, not laboratory, conditions. The article evaluates the accuracy of determining the change in the position of a structure using IMSGeo under real measurement conditions.

### Automatic Monitoring System—IMSGeo

**Figure 1:**
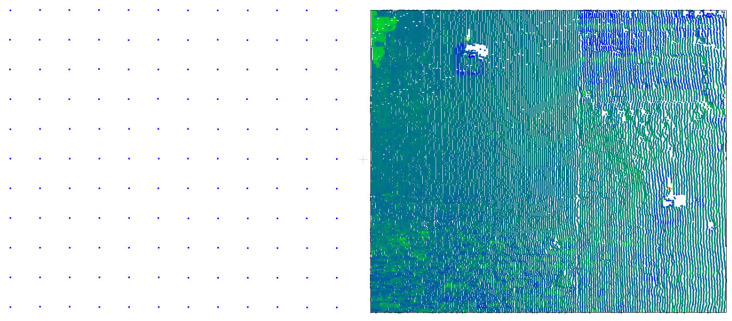
On the left, a set of points from the IMSGeo system (132 points) is shown; on the right, data from TLS for the analogous fragment of object–point cloud (42,852 points).

The IMSGeo system presented in the article compares data in point sets (X, Y, and Z) measured using reflectorless methods. These sets differ from point clouds obtained through terrestrial laser scanning (Figure 1) in the following aspects:

- The range of the scanned surface is determined based on the measurement of points with known coordinates;
- The distances between the measured points on the object are defined before the measurement; thus, their density is adjusted to the expected magnitude of change, which significantly reduces the size of the data sets;
- The points are measured in a regular grid;
- The point cloud is linked to a network of reference points and control points and thus is automatically oriented in the object/external coordinate system;
- The measurement to points is more accurate because it is performed using a motorized total station.

Terrestrial laser scanning is a relatively expensive technology. In monitoring systems for endangered objects, the use of TLS instruments is very rare. This is due to many factors related to the measurement method (rotating mirrors emitting and receiving a laser signal), the problem of measurement automation, and measurement errors. Monitoring with the use of laser scanners is carried out mainly for land deformation. The IMSGeo system uses a much cheaper solution for surface monitoring in the form of RTS instruments, which can not only measure the surface but also measure the control network on the object.

Monitoring systems for vulnerable structures must provide measurement data continuously or at intervals, depending on the dynamics of the changes occurring. This implies that one of the key aspects of selecting a system is the time required to acquire and interpret the data. This time depends on the measurement method, which directly correlates with the achieved accuracy, the amount of data collected, and the data processing method. In developing the IMSGeo system, the research team determined that using motorized total stations was optimal and proposed a two-step approach to data processing. The first step identifies whether the structure’s geometry changes, and the second quantifies the change. In both stages, the assessment of surface changes is based on determining the difference in normal vectors between successive measurement cycles. In the first step, this determination is made for the entire measured surface; if changes are detected, the subsequent step focuses on the designated areas. The decision to use normal vectors was based on the following:

- Measuring a deforming surface without a reflector, we will not always measure exactly at the same point;
- In some measurement cycles, we may not register all points in each field;
- The change based on normal vectors is very quickly determined and is used for the indication of a trend/situation in which something is happening on a given wall; we process data in groups, but we store each point in the database.

**Figure 2:**
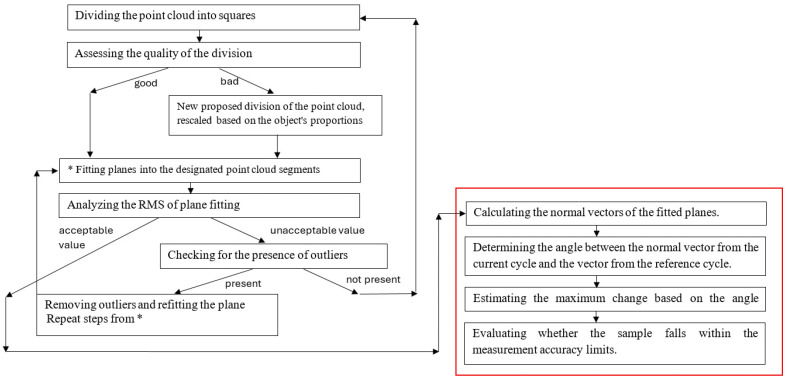
The consecutive steps of the cluster algorithm for determining maximum changes based on point clouds [26].

In the second step, an original clustering algorithm was used to process the data to quantify the change. The algorithm is presented as a decision diagram (Figure 2). A clustering algorithm is a way to divide and compare data to detect surface changes based on recorded point cloud data. The algorithm includes eight fundamental steps, which, in the case of a negative assessment of the results at any stage, are supplemented with an appropriate course of action. In the first step, the point clouds are divided into squares based on the reference measurement (structure before the change) and the subsequent one (performed to identify potential changes). If such a division cannot be made, separating the maximum number of squares and rectangles from the remaining part of the point cloud is recommended. The basis for choosing the size of the squares is the scanned surface’s vertical and horizontal dimensions, its type, and the expected change value. For flat, smooth surfaces where we expect, for example, deflections, we can apply larger squares than for rough, undulating surfaces, where changes occur suddenly and randomly. The quality of the division into groups is assessed visually, regarding the optimal fit of the squares. Outlier points are eliminated using statistical filters (e.g., S.O.R.). In Figure 2, the red color denotes the part of the clustering algorithm that allows for a quick assessment of whether there is any change in the structure’s geometry. A detailed assessment of the magnitude of the change should be carried out based on the entire procedure, with each step and an example of the analysis described in [26].

## 2. Materials and Methods

The article evaluates the accuracy of determining the change in the position of a structure using the clustering algorithm under real measurement conditions. The values determined by the algorithm result from fitting a plane to the specified cluster (a selected part of the recorded point cloud) using the least squares method with the normal equation of the plane and analyzing the normal vectors of the fitted plane.

A key element of the algorithm is determining the angle between the normal vector in the current measurement cycle and the corresponding normal vector from the reference (initial) cycle:

(1) $$cos\alpha =\frac{N_{Xr}*N_{Xi}+N_{Yr}*N_{Yi}+N_{Zr}*N_{Zi}}{\sqrt{N_{Xr}*N_{Xr}+N_{Yr}*N_{Yr}+N_{Zr}*N_{Zr}}*\sqrt{N_{Xi}*N_{Xi}+N_{Yi}*N_{Yi}+N_{Zi}N_{Zi}}},$$

where α represents the sought angle between the normal vectors, N denotes the components of the normal vector along the X, Y, and Z axes, with index r indicating the reference cycle, and index i indicating the current cycle.

Based on the angle between the normal vectors, the maximum possible value of the change in the position of points within the analyzed surface (∆dmax) is then determined:

(2) $$∆dmax=a*\left(b-1\right)*sin\left(\alpha \right),$$

where a is the distance between the measured points, b is the number of points in a single row of measurement points defined for the test field (Figure 1), and α is the angle between the normal vectors determined from the current cycle and the reference cycle data.

**Figure 3:**
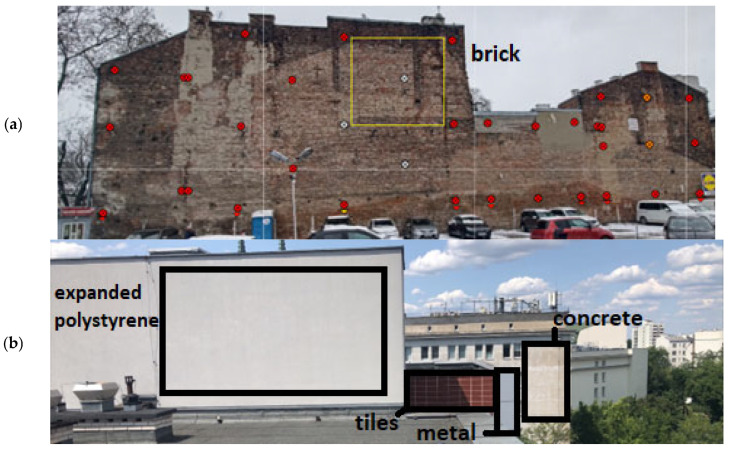
(**a**) View of the brick-surfaced structure on Stefan Okrzei Street in Warsaw. The monitored area is marked with a yellow frame. (**b**) View of structures representative of metal, tiled, polystyrene-insulated, and concrete facades on Stefan Okrzei Street in Warsaw.

The accuracy of determining the deformation of the structure based on the IMSGeo system was verified in two aspects: the repeatability of measurement results and the repeatability of results processed by the IMSGeo clustering algorithm. Five surfaces representative of construction structures’ elevation covers were selected for verification, measured under real conditions (Figure 3). For users, the key element is the accuracy and reliability of the system’s performance in field conditions. The developed monitoring system is based on a reflectorless tachymetric measurement, which has been thoroughly described in the literature based on studies in both laboratory and real conditions [27,28,29]. The presented experiment focused on globally verifying the accuracy of the algorithms’ performance in the IMSGeo system, rather than the instrument or reflectorless measurement technology.

To confirm the repeatability of measurement results for each surface, a comparison of the coordinates of all points X_i_, Y_i_, and Z_i_ from successive measurement cycles was performed.

Subsequent stages of development:

1. Selection of the reference measurement (initial “zero” measurement or another measurement cycle we take as reference).
2. Determination of the coordinate differences dX_i_ = X_r_ − X_i_, dY_i_ = Y_r_ − Y_i_, and dZ_i_ = Z_r_ − Z_i_, where X_r_, Y_r_, and Z_r_ are coordinates from the reference cycle, and X_i_, Y_i_, and Z_i_ are coordinates from the compared measurement cycle, as well as the difference in point distances ${dD}_{i}=\sqrt{{dX}_{i}^{2}+{dY}_{i}^{2}+{dZ}_{i}^{2}}$.
3. Determination of measurement error values according to the parameters of the instrument used and determination of the value of the mdi (distance measurement error) depending on the target length.
4. Determination of the mean error values of the measured points for the individual components $m_{X_{i}}$, $m_{Y_{i}}$, and $m_{Z_{i}}$ and the point position error value $m_{P_{i}}$ based on the formulas:

(3) $$\begin{array}{ll} m_{X_{i}}= & \sqrt{m_{X_{st}}^{2}+{\left(cosA_{P}*m_{d}\right)}^{2}+{\left(d_{st-i}\;*sin\;A_{P}*m_{A_{P}}\right)}^{2}},\\ m_{Y_{i}}= & \sqrt{m_{Y_{st}}^{2}+{\left(sinA_{P}*m_{d}\right)}^{2}+{\left(d_{st-i}\;*cos\;A_{P}*m_{A_{P}}\right)}^{2}},\\ m_{Z_{i}}= & \sqrt{m_{∆hi}^{2}+m_{i}^{2}+m_{w}^{2}} \end{array}$$

where $m_{X_{st}}$ and $m_{Y_{st}}$ are the mean position errors of the station, $A_{P}$ is the azimuth to the analyzed point, $d_{st-i}$ is the horizontal distance between the given point and the station, $m_{A_{P}}$ is the azimuth mean error, m_i_ is the instrument height determination error, m_w_ is the target height determination error, and $m_{d}$ is the distance determination error, where

(4) $$d_{st-i}\approx Scos\beta ,\;m_{d}=\sqrt{{\left(cos\beta *\;m_{S}\right)}^{2}+{\left(S*sin\beta *m_{\beta }\right)}^{2}},$$

S is the slope distance between the given point and the station, β is the vertical angle for the given point, $m_{S}$ is the slope distance error, and $m_{\beta }$ is the vertical angle. The mean error of measurement of height difference $m_{{∆h}_{i}}$ between two points is as follows:

(5) $$m_{{∆h}_{i}}=\sqrt{{\left(sin\beta *m_{s}\right)}^{2}+{\left(S*cos\beta *m_{\beta }\right)}^{2}+{\left(\frac{S^{2}}{2R}m_{k}\right)}^{2}},$$

where $m_{k}$ is the mean error of the refractive index, and R is the Earth’s radius. The point position error value is as follows:

(6) $$m_{P_{i}}=\sqrt{m_{Xi}^{2}+m_{Yi}^{2}+m_{Zi}^{2}},$$

Determination of alerts for the absolute values of coordinate differences exceeding twice the corresponding mean error values is as follows:

(7) $$alert{dX}_{i}=2*\sqrt{m_{Xr}^{2}+m_{Xi}^{2}},\;alert{dY}_{i}=2*\sqrt{m_{Yr}^{2}+m_{Yi}^{2}},\;alert{dZ}_{i}=2*\sqrt{m_{Zr}^{2}+m_{Zi}^{2}},$$

(8) $$alertd_{i}=2*\sqrt{m_{Xr}^{2}+m_{Yr}^{2}{+m}_{Zr}^{2}+m_{Zi}^{2}{+m}_{Yi}^{2}+m_{Zi}^{2}},$$

To confirm the repeatability of the results processed by the IMSGeo clustering algorithm in successive measurement cycles, the values of normal vectors for the designated test fields were compiled and compared. Normal vectors were determined according to the procedure outlined in the diagram in Figure 2. The coordinate differences and RMS (root mean square) values were determined according to the formulas below.

(9) $$\begin{array}{ccc} deltaX_{S} & = & X_{Sr}-X_{Si},\\ deltaY_{s} & = & Y_{sr}-Y_{si},\\ deltaX_{s} & = & Z_{sr}-Z_{si}, \end{array}$$

(10) $${RMS}_{i}=\sqrt{\frac{1}{n}\sum_{i=1}^{N}{d_{ort}}^{2}},$$

(11) $$delta{RMS}_{i}=\sqrt{{\left({RMS}_{r}\right)}^{2}+{\left({RMS}_{i}\right)}^{2}},$$

where n is the number of points in the square, and $d_{ort}$ is the orthogonal distance of the point from the fitted surface.

## 3. Results

The measurement was conducted on 4–5 April 2023 and 28–30 June 2023, using a Leica TCRP1201+ R1000 instrument, (distributed by Leica Geosystems Sp. z o.o., Poland, manufactured by Leica Geosystems AG—Part of Hexagon, Switzerland) characterized by a measurement accuracy of 1” for direction and 2 mm + 2 ppm for distance in the reflectorless mode. The measurement was taken from a distance of over 60 m for brick and concrete surfaces, while for expanded polystyrene, tile, and metal surfaces, the measurement distance was approximately 15 m. Detailed measurement parameters are provided in Table 1. During the measurement, difficulties related to weather changes were encountered, and their impact is described in the summary of each measurement.

### 3.1. Verification of Measurement Result Repeatability

In the first step, the coordinates of the measured points were directly compared, and the distances between corresponding points were determined. The maximum differences reached 2 cm, but these were exceptional cases, probably related to a disturbance during the measurement, e.g., a moving crane or horizontal movement of construction equipment. For most points, the calculated measurement accuracy was within 5 mm. In the analysis, repeatability is the percentage of points for which the alert did not occur. The analysis included alerts for the absolute values of coordinate differences exceeding twice the corresponding mean error values. Table 2 presents the percentage repeatability of X, Y, and Z coordinate values and distance measurements for selected surfaces in successive measurement cycles, analyzed in individual measurement cycles.

**Figure 4:**
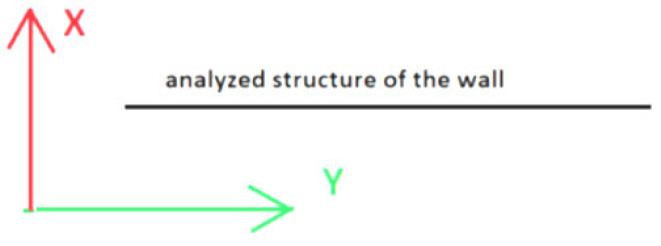
Method of adopting the local coordinate system relative to the analyzed surface.

Based on the results presented in Table 2, it can be concluded that the assumed 90% repeatability threshold was not achieved only for the y-coordinate. The error distribution theory justifies this situation. When points are parallel to one of the axes, the error value along that axis is most influenced by the angle measurement error, which is very small for the instrument used. Therefore, the coordinate determined along that axis is assumed to be measured with a small mean error. Furthermore, suppose the measurement repeatability is determined within the mean error of the coordinate determination. In that case, the repeatability criterion will be the strictest for the coordinate whose axis is parallel to the measured surface. Figure 3 shows the location of the measured surfaces in relation to the coordinate system. As can be seen, the situation described above concerned the y-axis, which in the IMSGeo system is always assumed to be parallel to the measured surface (Figure 4). As a result, by analyzing the maximum and minimum ${dY}_{i}$ values in the individual cycles for measurements where alerts occurred (calculated based on Formulas (7) and (8)), it was noted that their values did not exceed ±0.005 m (Table 3). In these cases, the calculated mean errors were around 0.0005 m, which triggered the alert after exceeding 1 mm (double the mean error value). Thus, the adopted criterion was very strict, considering the accuracy of the performed measurement. In Table 3, the measurement of the concrete surface on 29 June 2023 at 08:00 is highlighted in yellow, indicating significantly deviating results and suggesting a gross error. Based on the analysis, it was determined that the instrument had become damp during this measurement, and it was decided to exclude it from the overall summaries.

**Figure 5:**
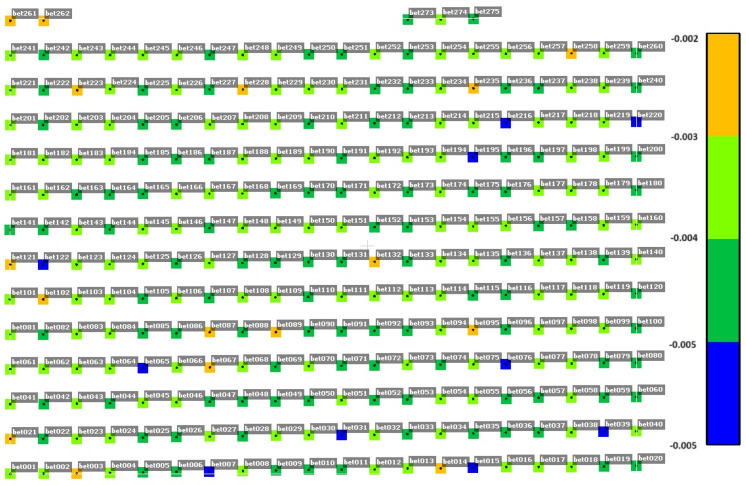
Distribution of ${dY}_{i}$ alert values for the concrete surface in the cycle on 28 June 2023, 18:00; repeatability 4%; Max [m] = −0.002; and Min [m] = −0.005.

**Figure 6:**
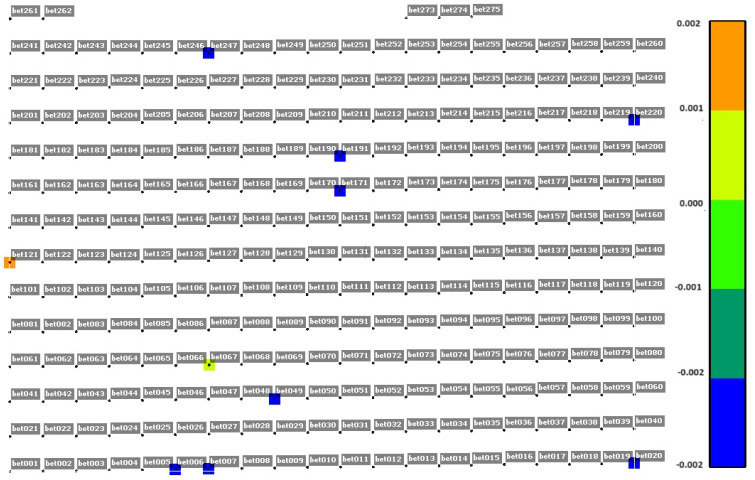
Distribution of ${dY}_{i}$ alert values for the concrete surface in the cycle on 28 June 2023, 22:00; repeatability 96%; Max [m] = 0.002; and Min [m] = −0.002.

**Figure 7:**
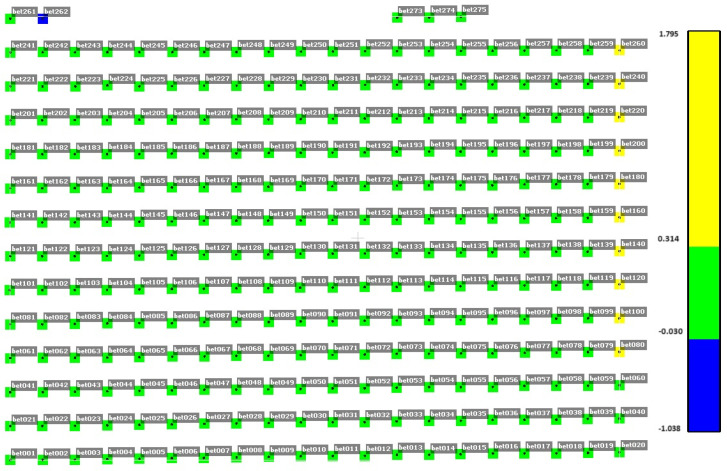
Distribution of ${dY}_{i}$ alert values for the concrete surface in the cycle on 29 June 2023, 08:00; repeatability 4%; Max [m] = 1.795; and Min [m] = −1.038.

To ensure that the occurrence of ${dY}_{i}$ coordinate alerts are not systematic, an analysis of the distribution of the y-coordinate difference in selected measurement cycles was performed for concrete surfaces (Figure 5, Figure 6 and Figure 7). Figure 5, Figure 6 and Figure 7 show the difference in ${dY}_{i}$ for individual points in selected measurement cycles. As can be seen, there is no repeatability for the change. The distribution of change magnitudes is random and indicates measurement errors caused by external factors.

Analyzing the data and considering the impact of the adopted coordinate system on the alert threshold values, it was determined that the key element confirming the high repeatability of measurements is the distance alert. This alert accounts for the spatial positioning of points in three dimensions. The distance alert demonstrated repeatability in the range of 96–100% for all measurement surfaces, which is considered a very good result given that the measurements were performed under real-world conditions.

The maximum distance values for individual points on selected surfaces are summarized in Table 4. The results indicate the presence of a few outlier points in the data set, which is consistent with the fact that a 100% repeatability was not achieved for all surfaces. However, in most cases, these differences were generally not significant.

### 3.2. Verification of Result Repeatability Using the Clustering Algorithm

In the second step of the analysis, the focus was on the repeatability of determining changes based on the clustering algorithm, which relies on comparing normal vectors. For all measurement cycles and the designated test fields, the differences in centroids ($deltaX_{S}$, $deltaY_{S}$, and $deltaZ_{S}$) and the differences in RMS values for plane fitting were compiled. The accuracy of the determined centroid coordinates and RMS values fluctuated around ±2 mm, with a few exceptions not exceeding ±5 mm, which is a very good result. The maximum distance differences between the reference plane and the planes determined in successive measurement cycles were also compared (Table 5).

**Figure 8:**
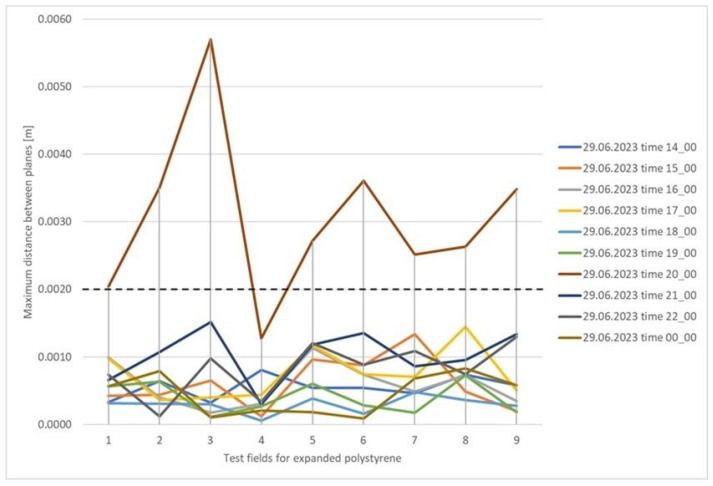
Graph of the maximum distance between the determined surfaces for individual test fields in successive measurement cycles for expanded polystyrene.

The maximum differences between the planes did not exceed 2 mm for brick and metal. For expanded polystyrene, 89% of the determined values did not exceed 2 mm. In this case, the exceedances occurred within a single measurement, during which a disturbance must have occurred, as shown in Figure 8.

**Figure 9:**
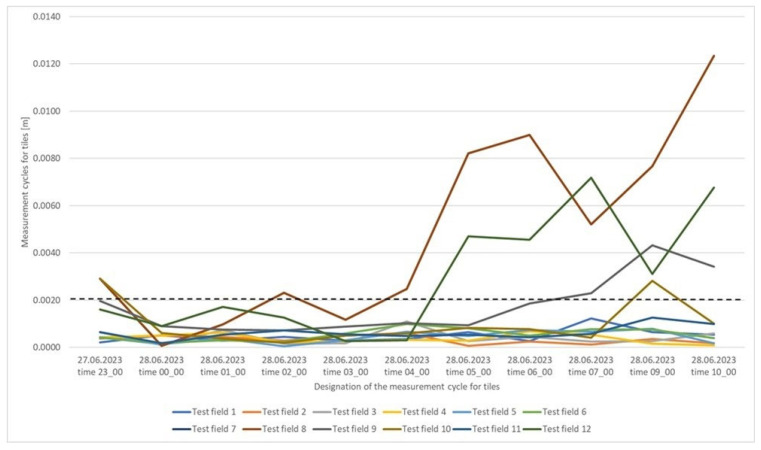
Graph of the maximum distance between the determined surfaces divided by test fields for individual measurement cycles for the tile surface.

**Figure 10:**
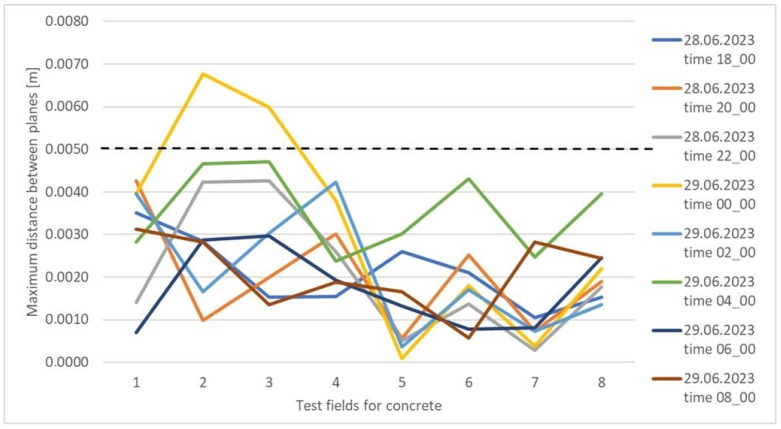
Graph of the maximum distance between the determined surfaces for individual test fields in successive measurement cycles for the concrete surface.

For tiles, 84% of the determined values did not exceed 2 mm (Figure 9). The figure shows that differences exceeding 2 mm occurred within four test fields, 8, 9, 10, and 12. Analyzing Table 5, the worst results were obtained for the concrete surface, where the maximum distance differences between the reference plane and the planes determined in successive measurement cycles did not exceed 5 mm in 97% of the cases. This is a good result considering the instrument’s accuracy and the characteristics of the measured surface, although it is worse than for the other surfaces. Figure 10 presents a concrete surface graph showing the maximum distance between the determined surfaces for individual test fields in successive measurement cycles. Based on the graph, it can be concluded that the greatest range of values occurs for fields 1–4, while fields 5–8 exhibit a consistent trend in most measurement cycles. It is worth noting that the concrete surface was divided into eight fields, with four fields per row, and the numbering started from the bottom left corner. Fields 1–4 indicated on the graph are on one level, while fields 5–8 are on a higher level. This suggests a certain dependency, which, given the accuracy of the measuring instrument, is classified as within the acceptable measurement error range.

The measurements for the analysis were conducted under real-world conditions on actual surfaces. This resulted in various factors, including weather, physical properties, and the location of the surfaces, which should be considered independent of the performers. However, the results obtained were very accurate.

Based on the abnormal measurements, conclusions were drawn regarding the protection of the instrument and the limitations in the precision of determining changes for surfaces with reflective elements, such as tiles or metal.

## 4. Discussion

In the conducted study, the repeatability of the obtained results was analyzed for raw (unfiltered) data. When comparing point to point for signaled measurements, there is no need for data filtration and special preparation. The IMSGeo monitoring system relies on normal vectors determined from a group of points. Fitting a plane to the point cloud relies on the normal distribution of distances, and each time the change in the structure will be averaged. Therefore, if there is an erroneous measurement in the group of points, it will affect the accuracy of the analysis. However, it should be noted that when analyzing sets containing dozens of points, minor measurement errors will not significantly impact the accuracy of determining the normal vector.

Using normal vectors, despite causing some averaging, is safer than directly comparing points, especially when comparing unsignaled points measured automatically and reflectorlessly, as we cannot be sure that the measurement was taken at exactly the same point on the measured surface. The results of our study have confirmed this. The maximum distance differences for individual points were the lowest for the metal surface −0.004 m, while the highest were for tiles −0.022 m. While the maximum distance between the determined surfaces divided by test fields for individual measurement cycles for metal did not exceed 0.002 m and, for the tile, −0.012 m. As can be seen, the results obtained using normal vectors are twice as good as those obtained with direct point-to-point measurement. All analyses achieved the highest agreement for the metal surface due to its low roughness. For the brick and tile surfaces, point-to-point comparisons yielded significantly worse results than analyses using the presented algorithm, which is consistent with the higher roughness of these surfaces. It should be emphasized that the measured surfaces were not deformed during the test measurements. However, the point-to-point comparison of the concrete surface resulted in alerts indicating significant exceedances of the permissible distance differences. The same area analyzed using normal vectors showed nothing concerning, consistent with the actual state. Outlying measurements were averaged.

It could be argued that in such a situation, we could average the characteristics of the actual deformation occurring in the structure. It should be remembered that deformations are changes in an entire area, and in the IMSGeo system, we measure a dense grid of points. Therefore, the key to correctly detecting changes using normal vectors is the proper selection of measurement parameters (point density depending on the expected change) and the appropriate selection of the size of the fields for which normal vectors are determined.

## 5. Conclusions

In the article, an analysis of the repeatability of measurement results was performed. For each of the five types of surfaces, measurements were obtained with very high accuracy and repeatability. The maximum differences in the coordinates of the corresponding points did not exceed 5 mm. For each of the five types of surfaces, the distance alert, which considers the location of points in three-dimensional space, ranged from 96% to 100%. The maximum differences in the distance between the reference plane and the planes determined in subsequent cycles were as follows: For bricks and metal, they did not exceed 2 mm; for tiles, 84% of the determined differences did not exceed 2 mm; and for expanded polystyrene, 89% did not exceed 2 mm; whereas for concrete, 97% of the determined differences did not exceed 5 mm.

The study shows the significance of properly securing the instrument and monitoring the conditions in which the measurement is performed. Additionally, it shows the significance of the properties of the measured surface, including its roughness and reflectivity.

## Figures and Tables

**Table 1:** Measurement parameters for various surfaces.

Surface Type	Number of Measurement Cycles/Time Interval Between Cycles	Distance Between Measurement Points	Surface Dimension: In Vertical and Horizontal Direction	Distance from Which the Measurement was Taken	Number of Measured Points in the Set
Brick	10/1 h	0.400 m	7.6 m/8.0 m	63 m	420
Metal	8/1 h	0.055 m	0.5 m/0.5 m	16 m	109
Tiles	12/1 h	0.080 m	0.7 m/2.3 m	16 m	300
Expanded polystyrene	11/1 h	0.140 m	1.7 m /2.2 m	15 m	285
Concrete	9/2 h	0.095 m	1.8 m/1.3 m	63 m	275

**Table 2:** Percentage repeatability of X, Y, and Z coordinate values and distance measurements for selected surfaces in successive measurement cycles.

Repeatability [%]	X_i_	Y_i_	Z_i_	D_i_
Brick	100%	9–99%	100%	100%
Metal	100%	8–99%	100%	100%
Tiles	97–100%	1–100%	98–100%	98–100%
Expanded polystyrene	96–100%	80–100%	94–100%	96–100%
Concrete	97%	4–100%	100%	99–100%

**Table 3:** Summary of the percentage repeatability of results within the accepted dYi alert threshold; maximum and minimum dYi alert values in the given measurement cycle for the analyzed surfaces.

**Tiles**							
Repeatability %	100	100	100	100	100	100	97
Max [m]	0.000	0.000	0.000	0.000	0.000	0.000	0.001
Min [m]	0.000	0.000	0.000	0.000	0.000	0.000	−0.001
Repeatability %	97	7	1	98			
Max [m]	0.001	0.001	0.002	0.001			
Min [m]	−0.001	0.001	0.001	−0.001			
**Brick**							
Repeatability %	97	99	98	98	98	51	89
Max [m]	−0.001	−0.001	0.002	0.002	0.002	0.003	0.003
Min [m]	−0.002	−0.002	−0.002	0.002	−0.001	0.001	0.001
Repeatability %	28	9					
Max [m]	0.004	0.005					
Min [m]	0.001	0.001					
**Concrete**							
Repeatability %	4	26	96	84	33	95	64
Max [m]	−0.002	−0.001	0.002	−0.001	−0.001	−0.001	−0.001
Min [m]	−0.005	−0.004	−0.002	−0.003	−0.003	−0.002	−0.003
Repeatability %	4						
Max [m]	1.795						
Min [m]	−1.038						
**Metal**							
Repeatability %	99	8	12	8	9	8	99
Max [m]	−0.001	0.002	0.001	0.002	0.001	0.002	0.001
Min [m]	−0.001	0.001	0.001	0.001	0.001	0.001	0.001
**Expanded polystyrene**							
Repeatability %	100	100	100	100	100	99	80
Max [m]	0.000	0.000	0.000	0.000	0.000	−0.002	−0.003
Min [m]	0.000	0.000	0.000	0.000	0.000	−0.002	−0.004
Repeatability %	100	100	93				
Max [m]	0.000	0.000	−0.002				
Min [m]	0.000	0.000	−0.003				

**Table 4:** Maximum distance values of individual points for selected surfaces.

Type of Surface	Max [m]
Expanded polystyrene	0.009
Metal	0.004
Concrete	0.009 (1.797—outlier)
Brick	0.011
Tiles	0.022

**Table 5:** Percentage of test fields for which the maximum distance differences between the reference plane and the planes determined in successive measurement cycles met the criteria.

Type of Surface	Percentage of Test Fields for Which the Given Criterion Was Met
Brick	100% did not exceed 2 mm
Metal	100% did not exceed 2 mm
Tiles	84% did not exceed 2 mm
Expanded polystyrene	89% did not exceed 2 mm
Concrete	97% did not exceed 5 mm

## Data Availability

The raw data supporting the conclusions of this article will be made available by the authors on request.

## Abbreviations

The following abbreviations are used in this manuscript:

ALS	Airborne Laser Scanning
GNSS	Global Navigation Satellite System
IMSGeo	Intelligent Monitoring System for Threatened Objects based on Automatic Non-invasive Measurements
RMS	Root Mean Square
RTS	Robotic Total Stations
SHM	Structural Health Monitoring
TLS	Terrestrial Laser Scanning
UAV	Unmanned Aerial Vehicle

## References

[1] Garbrecht G. (1986). Wasserspeicher (Talsperren) in der Antike. Antike Welt.

[2] Bauer P., Lienhart W. (2023). 3D concept creation of permanent geodetic monitoring installations and the a priori assessment of systematic effects using Virtual Reality. J. Appl. Geod..

[3] Bąk M. (2022). The use of automatic measurement techniques in the geotechnical monitoring system of PGE GiEK S.A., KWB Turów branch. Int. J. Coal Sci. Technol..

[4] Lubej S., Kovačič B. (2021). A Comparative Study of Signal Processing Methods for Contactless Geodetic Monitoring. Appl. Sci..

[5] Wiget A., Sievers B., Walser F. (2023). Contributions of geodesy to the safety of dams in Switzerland. Role of Dams and Reservoirs in a Successful Energy Transition.

[6] Li Y., Liu P., Li H., Huang F. (2021). A Comparison Method for 3D Laser Point Clouds in Displacement Change Detection for Arch Dams. ISPRS Int. J. Geo-Inf..

[7] Qian H. (2021). Design of Tunnel Automatic Monitoring System Based on BIM and IOT. J. Phys. Conf. Ser..

[8] Zhou J., Xiao H., Jiang W., Bai W., Liu G. (2020). Automatic subway tunnel displacement monitoring using robotic total station. Measurement.

[9] Hu D., Zhou R., Xiao L., Liang X., Li Y., Yang X. (2024). A Supershallow Buried Large-Span Rectangular Pipe Jacking Tunnel Undercrossing an Expressway: Construction Method, Monitoring Results, and Numerical Simulation. Int. J. Civ. Eng..

[10] Zaczek-Peplinska J., Kowalska M.E. (2022). Application of non-contact geodetic measurement techniques in dam monitoring. Arch. Civ. Eng..

[11] Zaczek-Peplinska J., Podawca K., Karsznia K. (2018). Reliability of geodetic control measurements of high dams as a guarantee of safety of the construction and the natural environment. Bull. Pol. Acad. Sci. Tech. Sci..

[12] Karsznia K., Zaczek-Peplinska J., Łapiński S., Odziemczyk W., Piasta Ł., Saloni L. The functionality assessment of geodetic monitoring systems for analyzing structural elements. Proceedings of the XXVII FIG Congress “Volunteering for the Future—Geospatial Excellence for a Better Living”.

[13] Galan-Martin D., Marchamalo M., Martínez R., Sanchez Sobrino J. (2013). Geomatics applied to dam safety DGPS real time monitoring. Int. J. Civ. Eng..

[14] Teng J., Shi Y., Wang H., Wu J. (2022). Review on the Research and Applications of TLS in Ground Surface and Constructions Deformation Monitoring. Sensors.

[15] Keyvanfar A., Shafaghat A., Awanghamat M.A. (2022). Optimization and Trajectory Analysis of Drone’s Flying and Environmental Variables for 3D Modelling the Construction Progress Monitoring. Int. J. Civ. Eng..

[16] Arif F., Khan W.A. (2021). Smart Progress Monitoring Framework for Building Construction Elements Using Videography–MATLAB–BIM Integration. Int. J. Civ. Eng..

[17] Ren D., Kang C., Peng T., Li Y., Wang J. (2024). Deformation Behavior of a Large-Scale Excavation and the Effect of an Adjacent Foundation Pit on the Excavation. Int. J. Civ. Eng..

[18] Kawajiri S., Hikichi S., Kondo K., Kagamihara S., Abe Y., Koizumi K. (2023). Observation of Pier Inclination Caused by Scouring Phenomenon of Foundation Ground Using a Large-Scale Open Channel and Development of Its Monitoring System. Int. J. Civ. Eng..

[19] Zacchei E., Lyra P.H.C., Lage G.E., Antonine E., Soares A.B., Caruso N.C., de Assis C.S. (2023). Structural Health Monitoring and Mathematical Modelling of a Site-Specific Concrete Bridge Under Moving Two-Axle Vehicles. Int. J. Civ. Eng..

[20] Pasternak G., Zaczek-Peplinska J., Pasternak K., Jóźwiak J., Pasik M., Koda E., Vaverková M.D. (2023). Surface Monitoring of an MSW Landfill Based on Linear and Angular Measurements, TLS, and LIDAR UAV. Sensors.

[21] Jäger R., González F. (2006). GNSS/LPS Based Online Control and Alarm System (GOCA)—Mathematical Models and Technical Realization of a System for Natural and Geotechnical Deformation Monitoring and Hazard Prevention. Geodetic Deformation Monitoring: From Geophysical to Engineering Roles.

[22] Zayats O. Automated System of Geodetic Monitoring. https://lpnu.ua/en/scientific-developments-directory/automated-system-geodetic-monitoring.

[23] Leica Geosystems AG Leica GeoMoS Monitoring Solution. https://leica-geosystems.com/en-gb/products/total-stations/software/leica-geomos.

[24] Topcon Delta LINK. https://www.topconpositioning.com/solutions/infrastructure/building-construction/monitoring.

[25] Trimble Inc. Trimble 4D Control. https://geospatial.trimble.com/en/products/software/trimble-4d-control.

[26] Kowalska M., Zaczek-Peplinska J., Piasta Ł. (2024). Determining the trend of geometrical changes of a hydrotechnical object based on data in the form of LiDAR point clouds. Arch. Civ. Eng..

[27] Fawzy H.E. (2015). Evaluate the accuracy of reflector-less total station. Int. J. Civ. Eng. Technol..

[28] Ford J. Effect of Environmental Surroundings on Accuracy and Precision of Total Station Measurements. Proceedings of the Engineering and Built Environment Project Conference 2015.

[29] Mohammed S.I. (2021). Important methods measurements to exam the accuracy and reliability of reflector-less total station measurements. J. Phys. Conf. Ser..

